# Essential metals at the host–pathogen interface: nutritional immunity and micronutrient assimilation by human fungal pathogens

**DOI:** 10.1093/femsyr/fov071

**Published:** 2015-08-04

**Authors:** Aaron Crawford, Duncan Wilson

**Affiliations:** Aberdeen Fungal Group, School of Medical Sciences, Aberdeen AB25 2ZD, UK

**Keywords:** zinc, iron, host–pathogen interactions, fungal pathogenicity

## Abstract

The ability of pathogenic microorganisms to assimilate sufficient nutrients for growth within their hosts is a fundamental requirement for pathogenicity. However, certain trace nutrients, including iron, zinc and manganese, are actively withheld from invading pathogens in a process called nutritional immunity. Therefore, successful pathogenic species must have evolved specialized mechanisms in order to adapt to the nutritionally restrictive environment of the host and cause disease. In this review, we discuss recent advances which have been made in our understanding of fungal iron and zinc acquisition strategies and nutritional immunity against fungal infections, and explore the mechanisms of micronutrient uptake by human pathogenic fungi.

## Abstract (precis)

The human body tightly sequesters essential micronutrients, restricting their access to invading microorganisms, and pathogenic species must counteract this action of ‘nutritional immunity’.

## Graphical abstract

**Graphical Abstract Figure.**
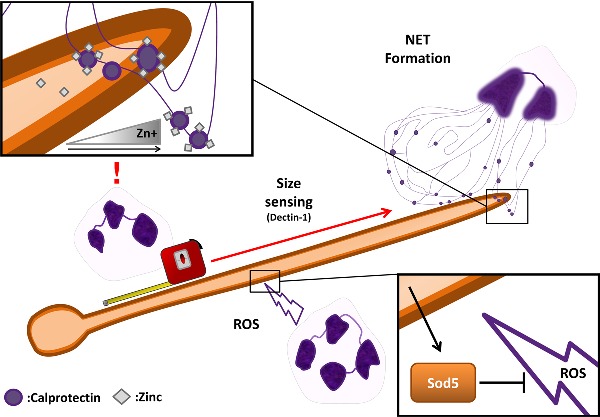
The human body tightly sequesters essential micronutrients, restricting their access to invading microorganisms, and pathogenic species must counteract this action of ‘nutritional immunity’.

## INTRODUCTION

Certain inorganic elements including iron, zinc and manganese are essential for life, and we need to obtain these ‘micronutrients’ in our diets. Pathogenic microorganisms are no exception, and must actively scavenge micronutrients from infected host tissue in order to grow. Our immune system has taken advantage of pathogens’ requirement for iron, zinc and manganese, by evolving sophisticated sequestration mechanisms to limit microbial access to these elements. Collectively, these processes of host-enforced micronutrient restriction are termed nutritional immunity (Hood and Skaar 2012). The role of (particularly iron-mediated) nutritional immunity in controlling bacterial infections is well established, and numerous bacterial iron uptake pathways have been shown to be essential for virulence (Hood and Skaar 2012). More recently, the wider importance of other transition metals (zinc, manganese and copper) in nutritional immunity has been established. For example, deletion of the high-affinity zinc import system in several bacterial pathogens results in attenuated virulence in relevant animal models (Hood and Skaar 2012).

As micronutrients are universally essential for life, many of the strategies the mammalian immune system has developed to control bacterial infections are also applicable to other pathogens. In this review, we discuss nutritional immunity in the context of fungal infections, which now account for an enormous burden on human health (Brown *et al.* 2012), and the mechanisms which human fungal pathogens have evolved to allow them to adapt to the nutritionally restrictive environment of the infected host.

### Haem iron utilization by pathogenic fungi

**Figure 1.**
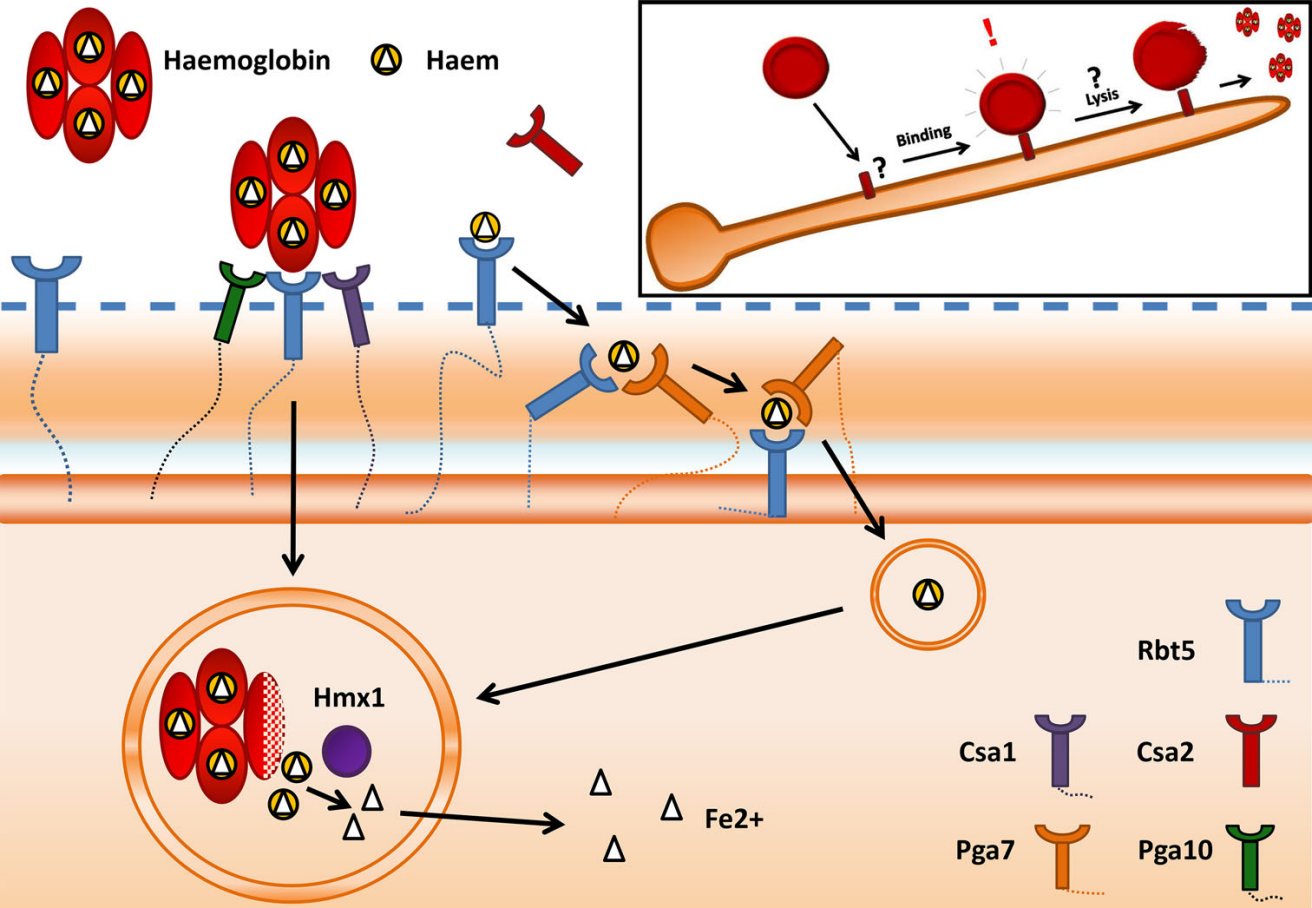
Model of haem/haemoglobin iron utilization by *C. albicans*. Erythrocytes are bound and lysed by *C. albicans* hyphae by as-yet unknown molecular mechanisms (inset). Released haem/haemoglobin is bound at the outer surface of the cell wall by members of the Rbt5 family and in the surrounding environment by the secreted Csa2. Haem is shuttled through the cell wall via an Rbt5-Pga7 relay network and endocytosed, before being metabolized in the vacuole by the haem oxygenase, Hmx1.

Iron is the most abundant transition metal within the human body, but free levels are maintained at extremely low levels, thus limiting availability to potential microbial pathogens. The majority of iron is complexed to haemoglobin and further segregated within erythrocytes. Therefore, to access this potentially rich iron source during infection, fungal pathogens must first lyse erythrocytes, bind haem/haemoglobin and then assimilate haem iron. This pathway has been most extensively investigated in *Candida albicans* (Fig. 1). Hyphae of *C. albicans* are able to ‘rosette’ complement-opsonized human erythrocytes, via an as-yet unknown surface receptor (Moors *et al.* 1992). Moors *et al.* proposed that this interaction was mediated by a fungal ligand with complement receptor-like properties, because rosetting of opsonized erythrocytes by *C. albicans* was blocked by an antibody against human CR3. Although complement-binding (MP60) and complement receptor 3-related (CR3-RP) proteins have been tentatively described in *C. albicans* (Eigentler *et al.* 1989; Stringaro *et al.* 1998), their genetic basis remains unsolved. On the other hand, the gene *PRA1* encodes a secreted protein with both factor H- and C3- binding properties. However, despite these various previous observations, the molecular basis for *C. albicans* binding of human erythrocytes remains undescribed.

Direct erythrocyte binding may facilitate closer proximity of the haemoglobin iron source; however, to then access it, the fungus must next permeabilize or lyse the erythrocyte membrane (Fig. 1, inset). Haemolytic activity has been described for *C. albicans* (Manns, Mosser and Buckley 1994); however, again, the molecular basis for erythrocyte membrane lysis has not yet been reported. Notably, *C. albicans* haemolytic activity was identified in the culture supernatants of hyphae, suggesting that the elusive haemolysin may be a secreted hypha-associated factor (Manns, Mosser and Buckley 1994). Following its release from erythrocytes, the fate of haemoglobin/haem iron assimilation by *C. albicans* has been characterized in excellent detail, primarily by the group of Kornitzer (2009). Haemoglobin/haem is primarily bound by members of the Rbt5 family of cell surface and secreted proteins (Fig. 1). *RBT5*, *PGA10*, *CSA1*, *CSA2* and *PGA7* each encodes proteins containing an eight-cysteine domain referred to as CFEM (*c*ommon in several *f*ungal *e*xtracellular *m*embrane proteins), and have been implicated in haem/haemoglobin binding in *C. albicans*. Rbt5 was initially identified via its ability to allow haemoglobin utilization by *Saccharomyces cerevisiae* and deletion of *RBT5* in *C. albicans* conversely reduced the ability of this fungus to utilize haemoglobin (Weissman and Kornitzer 2004). The current model of *C. albicans* haem iron assimilation (Fig. 1) is that haemoglobin/haem is bound by Rbt5 at the outer leaflet of the fungal cell and that, via a relay network involving Rbt5-Pga7, is passed through the cell wall (Kuznets *et al.* 2014) to the plasma membrane, where it is internalized via endocytosis (Weissman *et al.* 2008). Csa1 and Pga10 are also implicated in haem binding at the fungal cell surface and Csa2 is secreted to the extracellular environment where it may act as a ‘haemophore’ (Fig. 1). Finally, iron is released from internalized haem or haemoglobin via metabolism to α-biliverdin by the action of the haem oxygenase, Hmx1 (Pendrak *et al.* 2004).

Haem iron utilization by *C. albicans* appears to have direct clinical consequences. Rbt5 has been identified as an immunodominant antigen in the sera of candidaemia patients (Mochon *et al.* 2010), providing strong evidence that iron nutritional immunity does indeed occur in the clinical setting, and that *C. albicans* expresses its haem iron assimilation machinery during disseminated infection of a human host.

Haem iron assimilation is not limited to *C. albicans* and has been observed in other pathogenic species. *Paracoccidioides* also exhibits haemolytic activity and can utilize haemoglobin as an iron source. Indeed Bailão *et al.* (2014) identified a cell surface haemoglobin receptor in *Paracoccidioides* which facilitates internalization and utilization of haemoglobin-derived iron. Intriguingly, the identified protein shares similarities with *C. albicans* Rbt5 and possesses a CFEM motif. The *Paracoccidioides* Rbt5 was found to be essential for surviving macrophages and for virulence in a murine model of paracoccidioidomycosis (Bailão *et al.* 2014), indicating that haem-derived iron may represent the dominant iron source during *Paracoccidioides* infections.

The distantly related Basidiomycete human pathogen, *Cryptococcus neoformans* also expresses a cell surface haem-binding protein: Cig1. *CIG1* expression is regulated by iron availability and by the pH-responsive transcription factor, Rim101 (which, notably, regulates zinc acquisition in *Aspergillus fumigatus*, see below). Simultaneous deletion of *CIG1* and *CFO1* (encoding a ferroxidase of the high-affinity reductive iron uptake system) in *Cr. neoformans*-attenuated virulence in a mouse model of cryptococcosis, indicating that both iron uptake pathways (redundantly) operate during infection (Cadieux *et al.* 2013). Interestingly, despite their similar function, *Cr. neoformans* Cig1 does not appear to be phylogenetically related to the Rbt5 family of haem-binding proteins of *C. albicans* and lacks the typical CFEM motif. This suggests that the ability to exploit mammalian haem as an iron source is an independently evolved trait in these two major human fungal pathogens.

*Histoplasma capsulatum* has also been reported to utilize hemin via binding at the cell surface, by an as-yet uncharacterized mechanism. In contrast, the major human pathogenic mould, *A. fumigatus*, does not utilize haemoglobin or hemin (Schrettl *et al.* 2004); similarly, *C. glabrata* cannot utilize haem (Nevitt and Thiele 2011). Therefore, distinct human fungal pathogens (including the distantly related species, *C. albicans* and *Cr. neoformans*) have the potential to exploit haem-derived iron during infection; and it would appear that the ability to utilize mammalian iron-binding proteins has arisen more than once in the evolution of human fungal pathogens.

### Ferritin exploitation in the host

**Figure 2.**
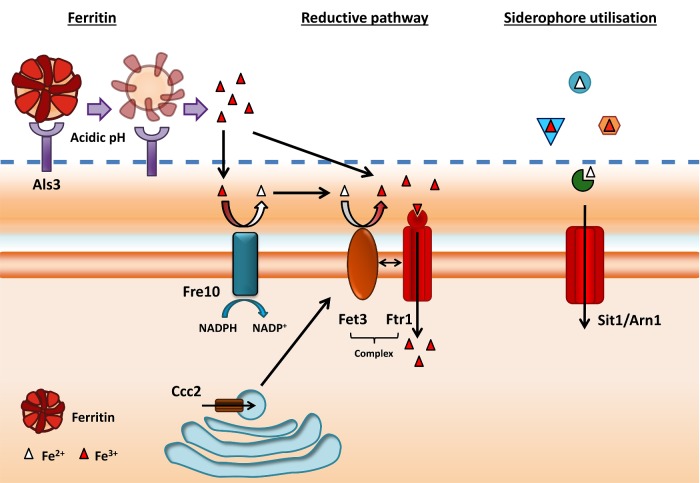
Model of ferritin and siderophore utilization by *C. albicans*. Ferritin is bound by Als3 at the hyphal cell surface. Local acidification releases ferric (Fe^3+^) iron which is reduced to ferrous (Fe^2+^) iron by the action of a family of ferric reductases (e.g. Fre10). Ferrous iron is subsequently reoxidized to ferric iron via multicopper oxidase activity (Fet3). Ferric iron is transported into the cell via the high-affinity permease, Ftr1. *Candida albicans* assimilates xenosiderophores (siderophores produced by other species) via the siderophore transporter, Sit1/Arn1.

Within tissue, iron is predominantly sequestered within the intracellular iron storage molecule, ferritin. This 24-subunit protein nanocage has the capacity to store up to 4500 ferric (Fe^3+^) ions, and thus represents a potentially lucrative nutrient source for intracellular or invasive fungal pathogens (Almeida, Wilson and Hube 2009). Indeed, the invasive hyphal morphology of *C. albicans* has been shown to bind host ferritin at the hyphal cell surface, both *in vitro* and during coincubation with human epithelial cells (Almeida *et al.* 2008) (Fig. 2). This binding event was found to be mediated by the hypha-associated cell-wall protein Als3, as deletion of *ALS3* in *C. albicans* precluded ferritin binding of hyphae and heterologous expression of *ALS3* in *S. cerevisiae* facilitated ferritin binding by this normally non-pathogenic yeast (Almeida *et al.* 2008). Following ferritin binding by *C. albicans* hyphae, subsequent assimilation and utilization of ferritin–iron requires both acidification of the surrounding environment (presumably to facilitate iron release, as ferritin is remarkably stable at physiological pH) and activity of the reductive iron assimilation pathway (Fig. 2). The reductive pathway itself takes place at the fungal plasma membrane and consists of a series of reactions involving multiple gene families. Ferric (Fe^3+^) iron is reduced to ferrous (Fe^2+^) iron by a family of ferric reductases. *Candida albicans* encodes at least 15 potential ferric reductase paralogues (Almeida, Wilson and Hube 2009). Next, the reduced ferrous iron is reoxidized by the action of multicopper oxidase. Resultant ferric iron is transported into the fungal cell via the high-affinity iron permease, Ftr1. Interestingly, deletion of *FTR1* in *C. albicans* renders the fungus avirulent in a murine model of disseminated candidiasis (Ramanan and Wang 2000), indicating that the reductive pathway may be essential for iron acquisition during systemic disease. This is in contrast to *A. fumigatus* where the reductive pathway is dispensable for virulence, and siderophore-mediated iron scavenging plays a crucial role during infection (see below).

*Knowing what to expect*. Notably, both the ferritin and haem-binding proteins of *C. albicans* (Als3 and Rbt5, respectively) are more highly expressed by the invasive hyphal morphology than by yeast cells (Braun *et al.* 2000; Nobile and Mitchell 2005). In this context, accumulating evidence suggests that in certain host niches (e.g. mucosal epithelia), yeast and hyphae represent, respectively, the commensal and pathogenic morphologies of *C. albicans* (Moyes and Naglik 2011; Moyes *et al.* 2011). Moreover, the yeast-to-hypha transition is intimately associated with invasive growth by *C. albicans* (Dalle *et al.* 2010; Wachtler *et al.* 2011; Mech *et al.* 2014). Therefore, it would appear that *C. albicans* may have ‘hardwired’ the expression of invasive-specific iron utilization pathways (Als3/ferritin & Rbt5/haem) into its hyphal morphogenic programme. This concept of ‘adaptive prediction’ describes the phenomenon whereby a microorganism ‘pre-expresses’ proteins of temporally subsequent relevance (Mitchell *et al.* 2009) and has recently been proposed for pathogenic fungi such as *C. albicans* (Brunke and Hube 2014). In this case, *C. albicans* may have ‘learned’ that the switch from yeast to hyphae, which is predicted to commit the fungus to invasive growth (Mech *et al.* 2014), is intimately associated in nature with the subsequent availability of intracellular iron sources, such as haemoglobin/haem and ferritin.

### Siderophore-mediated iron scavenging

Siderophores represent a highly effective iron acquisition mechanism. Many microbes, and some plants, produce high-affinity iron chelators (siderophores), which are released to the environment, sequester iron, and are then transported back into a cell for iron assimilation (Haas 2014). A number of human pathogenic fungal species can produce, or utilize exogenous siderophores. The common pathogenic mould, *A. fumigatus* produces both extracellular siderophores, which scavenge iron from the environment and the intracellular siderophores ferricrocin and hydroxyferricrocin for intracellular iron storage. Importantly, perturbation of siderophore biosynthesis (via deletion of *sidA*, which catalyses the first committed step in siderophore biosynthesis), precluded *A. fumigatus* virulence in a murine model of invasive aspergillosis (Schrettl *et al.* 2004). Interestingly, in the same study, Schrettl and co-workers found that, in stark contrast to *C. albicans* (Ramanan and Wang 2000), perturbation of reductive iron assimilation in *A. fumigatus* had no impact on virulence, and that *A. fumigatus* appears to lack uptake systems for host-specific iron sources, such as ferritin and haemoglobin. The latter observation may reflect the evolutionary adaptation of *A. fumigatus* to environmental niches, rather than to a human host.

*Candida albicans* does not synthesize its own siderophores, but can utilize those produced by other species (Fig. 2). These are known as ‘xenosiderophores’ and *C. albicans* can assimilate ferrichrome-type siderophores including ferricrocin, ferrichrysin, ferrirubin, coprogen and triacetylfusarinine C via the cell surface transporter, Sit1 (Heymann *et al.* 2002). Interestingly, *C. albicans* Sit1 was found to be required for invasion of a human epithelial multilayer, but was dispensable for virulence in a murine model of systemic candidiasis (Heymann *et al.* 2002). The Sit1 orthologue in *C. glabrata* has also been shown to play a role in the assimilation of the xenosiderophores, ferrichrome, ferrirubin and coprogen. Indeed, pulsing *C. glabrata* wild type, but not *sit1*Δ, cells with ferrichrome before subsequent macrophage exposure enhanced fungal survival (Nevitt and Thiele 2011). Although a controlled *in vitro* experiment, this chain of events may have clinical parallels.

In their natural environmental niches (including, e.g., the human gastrointestinal tract), colonizing *Candida* cells are exposed to numerous bacterial species of the microbiome. The ability to efficiently scavenge siderophore-derived iron in this setting may therefore prime fungal cells with intracellular iron storage, which can subsequently be utilized during invasive infections, where exogenous iron availability is highly limited. Indeed, it is likely that, in contrast to bacteria, the ability of fungal cells to store significant levels of micronutrients (particularly in the vacuole) may have far-reaching implications in studies on host–pathogen interactions.

The differential capacity of the major human pathogenic species to utilize differing iron sources as described above is in line with the concept of independently evolved virulence of human fungal pathogens (Bowman, Taylor and White 1992; Wilson *et al.* 2014) and it would appear that different human fungal pathogens utilize different strategies to secure iron from their hosts.

### Iron nutritional immunity during fungal infection

Despite the elemental importance of iron on the outcome of pathogenesis, global host nutritional immunity during fungal infections has only recently been investigated. Potrykus and co-workers used a powerful combination of laser ablation–inductively coupled plasma–mass spectrometry (LA-ICP-MS), MALDI imaging, immunohistochemistry and microtranscriptomics to dissect the impact of systemic candidiasis on both local and global iron homeostasis (Potrykus *et al.* 2013, 2014). They found that as *C. albicans* formed lesions in the renal cortex (a pathology typical for this form of candidiasis), iron was mobilized away from the fungal lesions to the renal medulla. This event was associated with increased levels of the host iron-binding proteins, ferritin and haemoglobin alpha in the medulla of infected kidneys, and the accumulation of haem oxygenase around the fungal lesions, indicating that the sequestration of iron away from the sites of infection is indeed a coordinated host-driven process, and likely represents the first demonstration of systemic nutritional immunity against a fungal infection. On the pathogen side, it would appear that *C. albicans* relies on the reductive iron assimilation pathway during the early stages of infection, whilst utilizing haem-derived iron sources during later phases of infection (Potrykus *et al.* 2013).

### Zinc sequestration by calprotectin

Whilst the host–pathogen struggle for iron has been characterized in greatest detail, the importance of other metals in nutritional immunity is beginning to be appreciated. Zinc is the second most abundant transition metal in the human body and, like iron, its availability is tightly regulated. The higher level hierarchy of zinc nutritional immunity is not as well understood as for iron; nevertheless, more recent studies have begun to elucidate zinc sequestration mechanisms at organism, organ, tissue and cellular levels, primarily in the context of bacterial infections (Kehl-Fie and Skaar 2010; Hood and Skaar 2012).

Against fungal pathogens, the dominant described mechanism of zinc restriction is via the action of the antimicrobial peptide, calprotectin. Calprotectin, also known as calgranulin, is a heterodimer composed of two subunits: S100A8 and S100A9. Following dimer assembly, calprotectin binds zinc (and manganese) with high affinity, limiting metal availability to microbes in the local environment (Corbin *et al.* 2008). Calprotectin accounts for a striking 45% of neutrophil cytoplasmic content (Edgeworth *et al.* 1991). Given the propensity for neutrophil infiltration during mycoses (e.g., mucosal candidiasis and invasive kidney colonization), the potential levels of calprotectin at sites of infection are therefore very high.

**Figure 3.**
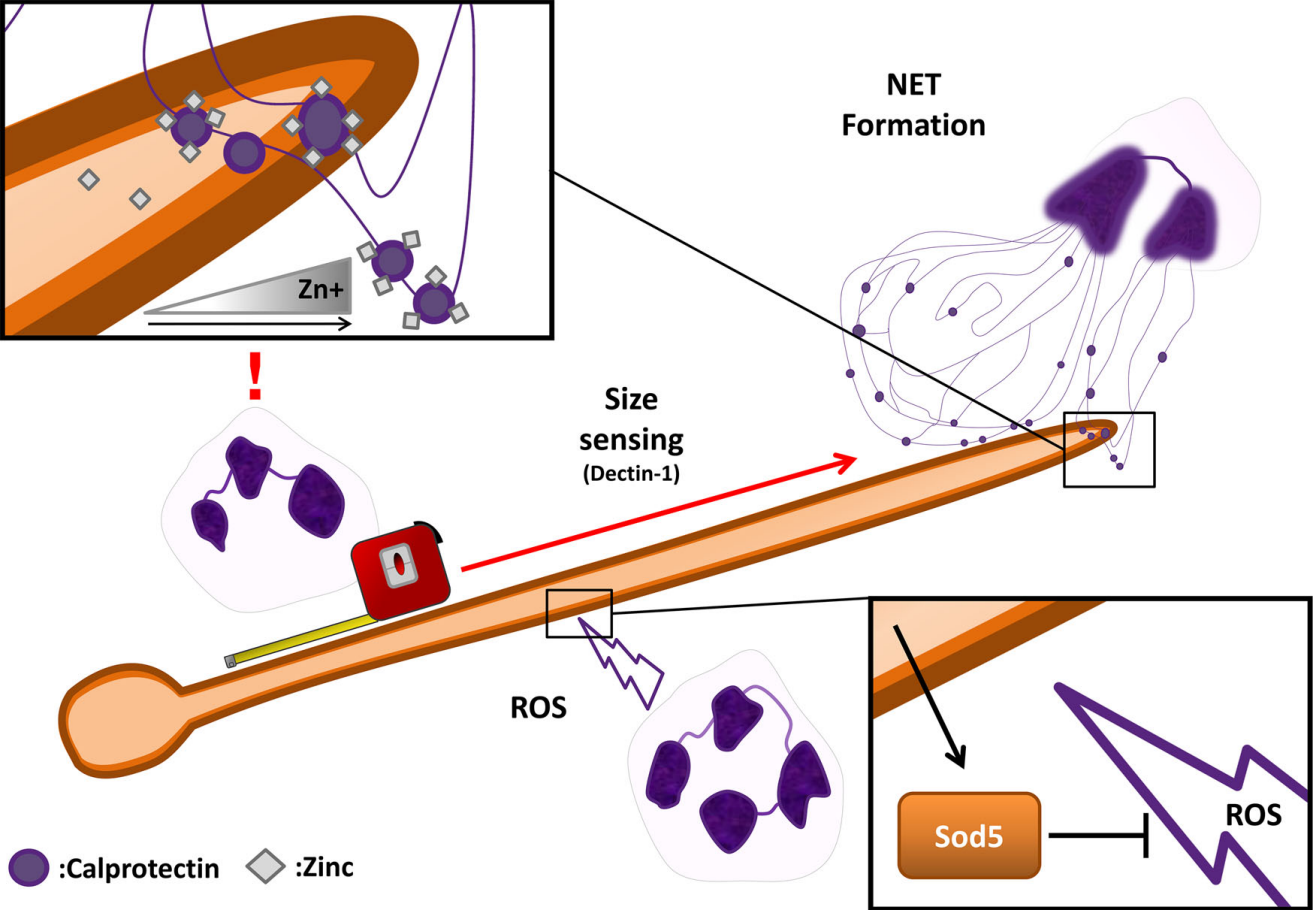
Model of the antifungal activity of neutrophil extracellular traps and calprotectin. Neutrophils sense the larger physical dimensions of pathogens such as *C. albicans* hyphae and undergo *NETosis*. The high levels of calprotectin decorating the NETs elicit local zinc depletion against the fungus (inset, top left). Viable neutrophils target the fungus with ROS; *C. albicans* counterattacks with expression of the copper-only superoxide dismutase, Sod5 (inset, bottom right).

The pathway of neutrophil–calprotectin antifungal activity is a fascinating and multifaceted process (Fig. 3). When neutrophils are appropriately stimulated, or when they sense large microbial structures, such as *C. albicans* hyphae, which may prove problematic to phagocytose, they can undergo a distinct form of programmed cell death known as *NETosis* (Brinkmann *et al.* 2004; Urban *et al.* 2006; Branzk *et al.* 2014). This involves coordinated chromatin decondensation, and the release of NETs (*n*eutrophil *e*xtracellular *t*raps). These spider web-like structures are composed of DNA, decorated with a limited number of associated proteins, including histones, lactoferrin, MPO, elastase and calprotectin (Urban *et al.* 2009). Scanning electron microscopy has revealed that NETs tightly associate with *C. albicans* cells and efficiently inhibit the growth of this fungus, as well as *Aspergillus* and *Cryptococcus* species (Urban *et al.* 2009).

Interestingly, the metal-sequestration properties of calprotectin appear to be the dominant mechanism of NET antifungal activity. Urban *et al.* (2009) demonstrated that treatment of digested NETs with anti- S100A8 and S100A9 antibodies abolished their antifungal activity and that NETs derived from calprotectin-deficient mice had significantly reduced activity compared to NETs from wild-type mice. In addition, treatment with excess zinc (and manganese) reversed NET inhibition of fungal growth (Urban *et al.* 2009). Growth of *Cr. neoformans* and *A. fumigatus* is also inhibited by calprotectin and NETs, suggesting broad-spectrum activity against human fungal pathogens (Urban *et al.* 2009; McCormick *et al.* 2010; Amich *et al.* 2014). Therefore, NET control of fungal growth via calprotectin-mediated sequestration of the essential micronutrients zinc and manganese represents a fascinating example of nutritional immunity being directed towards physically large pathogens, such as fungal hyphae (Fig. 3).

Importantly, continuous *C. albicans* hyphal cells can be simultaneously attacked by NETs and viable neutrophils (Urban *et al.* 2006). This is a significant observation because non-phagocytosed *C. albicans* cells in contact with neutrophils experience oxidative stress, likely due to the generation of extracellular reactive oxygen species (ROS) by the immune cell (Miramon *et al.* 2012). Therefore, during infection, *C. albicans* may concurrently experience zinc starvation and oxidative stress. This is an important combination of environmental insults because, as discussed below, an important element of the fungal oxidant detoxification system (superoxide dismutase) requires zinc for function (Hwang *et al.* 2002). Notably, Sod5, a ‘copper/zinc superoxide dismutase’ plays a crucial role in oxidative stress resistance and in surviving encounters with human neutrophils (Fradin *et al.* 2005). However, *C. albicans* appears to have evolved a novel method to circumvent the zinc-starvation/oxidative stress dilemma: Gleason *et al.* (2014) have recently shown that rather than requiring copper/zinc for function, *C. albicans* Sod5 represents a prototypic ‘copper-only’ superoxide dismutase (see below).

The two subunits of the calprotectin heterodimer, S100A8 and S100A9, belong to the S100A family of low molecular weight proteins. A third member of this family S100A7, also known as psoriasin, due to its heightened expression in psoriatic lesions, also exhibits antimicrobial activity via zinc sequestration (Glaser *et al.* 2005). Psoriasin is important for controlling microbial growth at body surfaces, such as the mucosae and, especially, the skin. Although most studies on psoriasin have focused on *Escherichia coli*, a recent report has described inhibition of dermatophyte growth by this antimicrobial protein (Fritz, Beck-Jendroschek and Brasch 2012). Although zinc binding has been reported for other members of the S100A family, their implications in controlling fungal infections have not yet been investigated.

### Zinc nutritional immunity in macrophages

So, the dominant mechanism of extracellular zinc depletion is via calprotectin; but what about intracellular fungal pathogens? Three of the major human pathogenic yeasts, *Cr. neoformans*, *C. glabrata* and *H. capsulatum* appear to experience prolonged periods of time within human immune cells, particularly macrophages. Indeed, it has been proposed that this intramacrophage stage may represent an important element of the pathogenic lifestyle of these yeasts, allowing them to evade other elements of host immunity (Seider *et al.* 2010; Miramon, Kasper and Hube 2013).

The fungal-containing phagolysosomes of macrophages likely represents a zinc-poor environment, evidenced by the fact that phagocytosed *C. albicans* cells upregulate expression of the zinc transporter-encoding gene, *ZRT2* (Lorenz, Bender and Fink 2004).

Indeed, intracellular zinc homeostasis within fungal-containing macrophages has recently been shown to be a highly dynamic and regulated process. Winters *et al.* (2010) originally described opposing actions of the two cytokines, GM-CSF and IL-4, on zinc homeostasis within *H. capsulatum*-containing macrophages. GM-CSF enhances the antimicrobial activity of macrophages, and inhibits the growth of phagocytosed *H. capsulatum* cells, whilst IL-4 conversely inhibits fungal killing. Using the highly sensitive metal quantification technique, ICP-MS, these authors found that GM-CSF caused a decrease in zinc availability to the phagocytosed yeast, whilst IL-4 treatment reversed this effect (Winters *et al.* 2010).

**Figure 4.**
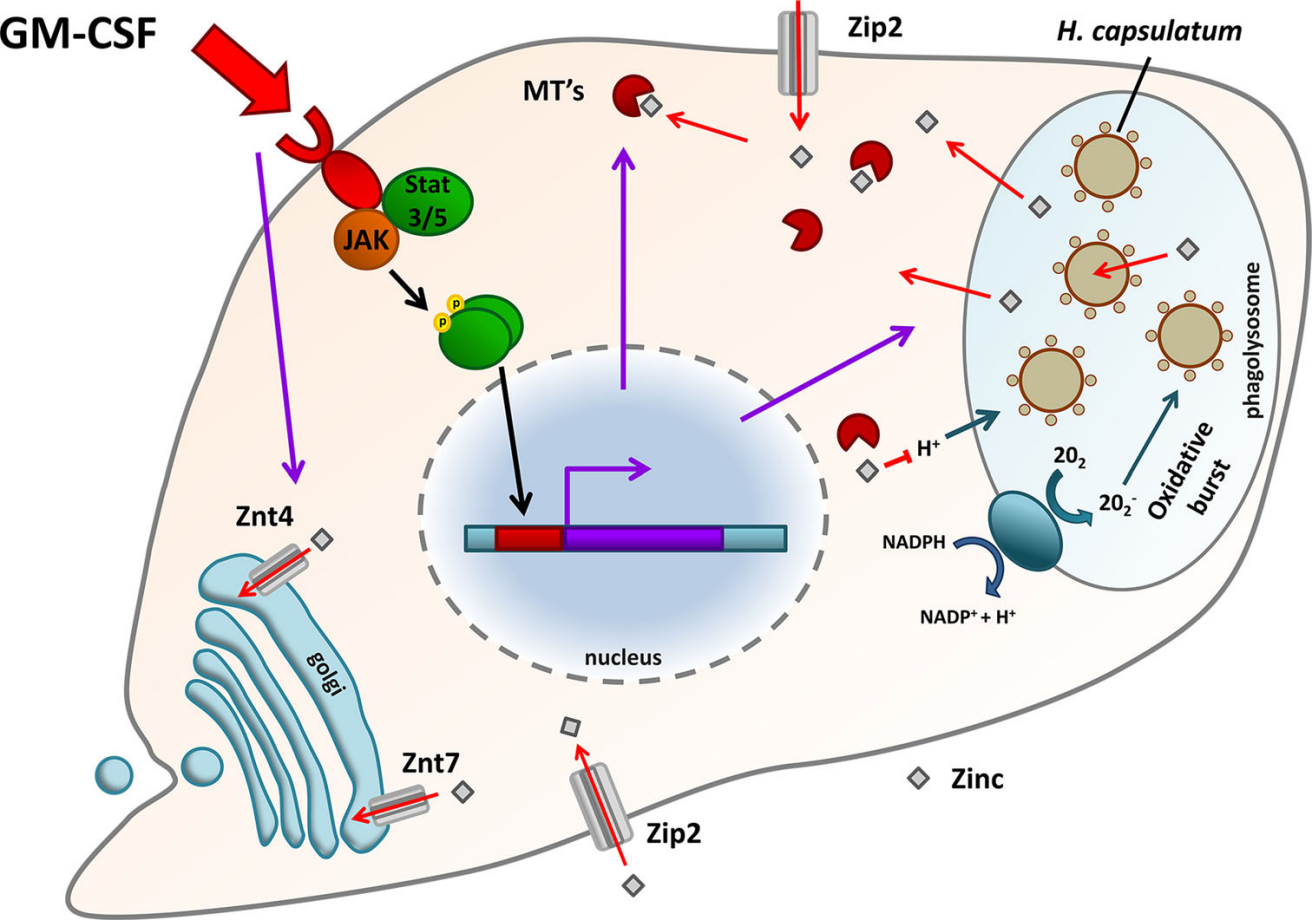
Zinc manipulation in activated *H. capsulatum*-containing macrophages. Activation of macrophages via GM-CSF results in STAT3/5-dependent upregulation of metallothioneins (MT), which bind zinc. Zinc is further sequestered away from phagocytosed yeast cells via the action of the ZnT-type zinc transporters, ZnT4 and ZnT7. Total cellular zinc is elevated via ZIP2. These events are associated with an enhanced oxidative burst.

More recently, the same group have elucidated the host cellular mechanisms which govern this form of ‘single-cell’ zinc nutritional immunity (Fig. 4). Subramanian Vignesh *et al.* (2013a) found that resting (unactivated) macrophages infected with *H. capsulatum* exhibited diffuse levels of zinc throughout the cell (visualized by staining with the zinc-specific probe, Zinpyr1), indicating that, in the absence of macrophage stimulation, *H. capsulatum* may have ready access to this key micronutrient. However, upon GM-CSF treatment, zinc was mobilized away from the phagocytosed yeasts and shuttled into the Golgi apparatus. This mobilization of macrophage zinc coincided with a decrease in *H. capsulatum* zinc availability. Intra-Golgi compartmentalization was probably due to action of the two ZnT-type zinc transporters ZnT4 and ZnT7, as the encoding genes, *Slc30a4* and *Slc30a7*, respectively, were upregulated in activated macrophages infected with *H. capsulatum*. This class of cation efflux protein transports zinc from the cytosol into subcellular organelles (or pumps the metal out of the cell) and both ZnT4 and ZnT7 had been previously implicated in Golgi zinc transport in other cell types (Gao *et al.* 2009; McCormick and Kelleher 2012). Within the macrophage, zinc availability was likely further restricted by the action of zinc-binding metallothioneins, as expression levels of *Mt1* and *Mt2* were increased in a STAT3/STAT5-dependent manner, concomitant with zinc restriction. These events were also associated with enhanced ROS production, creating a ‘perfect storm’ of antimicrobial activity (see below). Counterintuitively, in parallel with these zinc sequestration and compartmentalisation events, total zinc levels within activated/infected macrophages were actually higher than under control conditions. This was likely due to upregulation of the cellular zinc importer, ZIP2 (Subramanian Vignesh *et al.* 2013a,b). Increased total zinc load in these macrophages may be necessary to deal with the increased metabolic demand associated with clearing phagocytosed yeasts. Alternatively, increased zinc uptake by macrophages may form part of an immune strategy to deplete zinc from the extracellular environment, analogous to the hepcidin axis of extracellular iron depletion (Nairz *et al.* 2014).

### Zinc uptake mechanisms of human pathogenic fungi

We have discussed the sophisticated mechanisms of host-driven zinc sequestration; however, zinc is absolutely essential for fungal proliferation and, in spite of the activities of host nutritional immunity, pathogens are still able to thrive in the infected host. Therefore, species which have evolved pathogenic potential must also have evolved mechanisms to efficiently scavenge micronutrients under the restrictive conditions of nutritional immunity. In this following section, we discuss the fungal factors which permit zinc assimilation and growth within their mammalian hosts.

**Figure 5.**
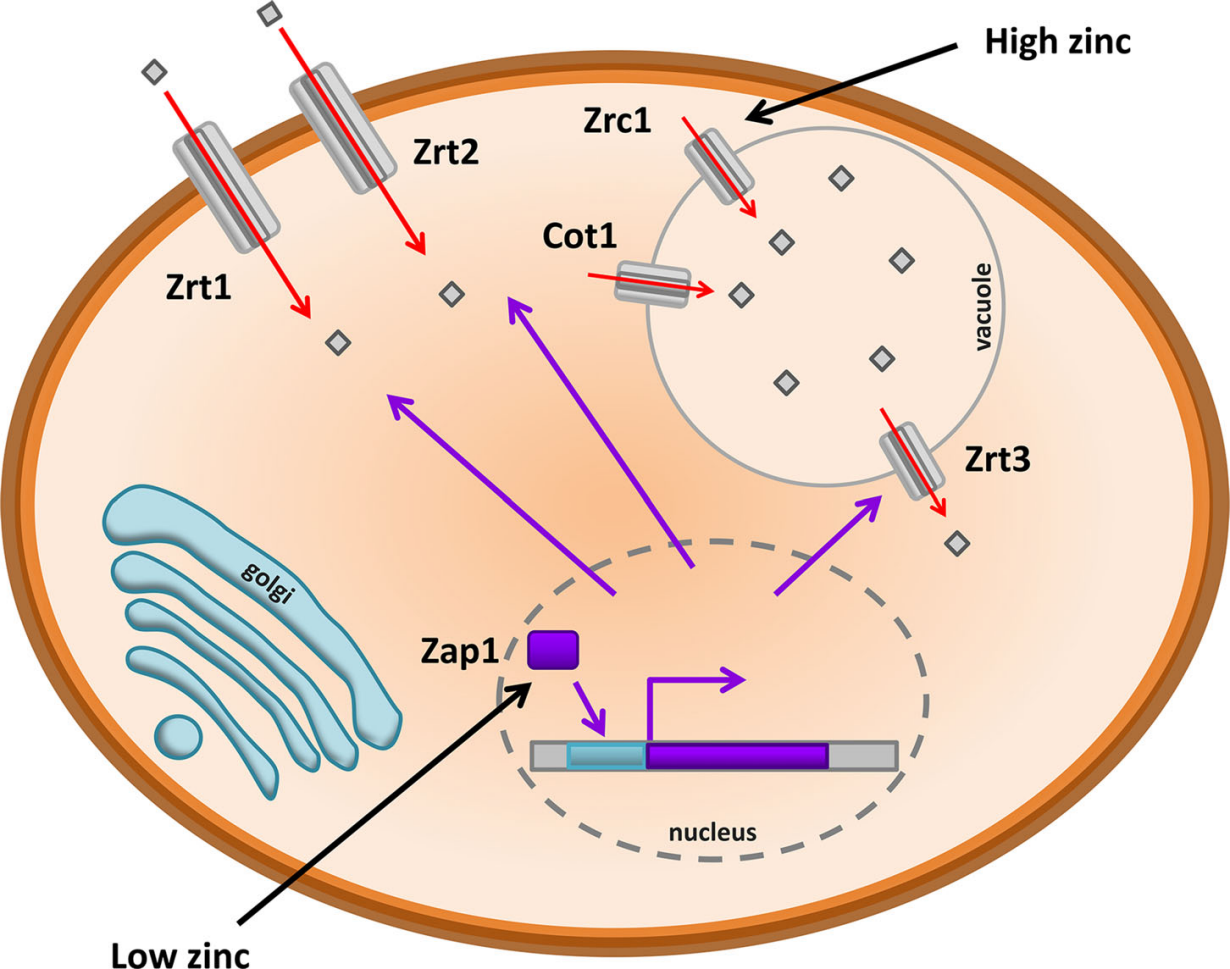
Model of zinc homeostasis in the model yeast, *S. cerevisiae*. The zinc-responsive transcription factor Zap1 responds to diminishing metal levels by triggering expression of the zinc importers, Zrt1 and Zrt2, and the vacuolar zinc exporter, Zrt3. In the presence of elevated zinc, the metal is efficiently detoxified in the fungal vacuole via Zrc1 and Cot1.

Although not a common human pathogen, studies in *S. cerevisiae* have laid the groundwork for our understanding of zinc transport in eukaryotic cells: both in mammalian systems and in other fungi (Fig. 5). This model yeast secures zinc from its environment via the action of two cell surface ZIP-type transporters: Zrt1 and Zrt2, which have high and low affinity for zinc, respectively (Zhao and Eide 1996a,b). Zinc transport in *S. cerevisiae* is governed by the zinc-responsive transcription factor, Zap1 (Zhao and Eide 1997). Following internalization, zinc can be shuttled to the vacuole by the ZnT-type transporters, Zrc1 and Cot1 (Kamizono *et al.* 1989; Conklin *et al.* 1992). Indeed, in yeast, this sequestration event can result in significant vacuolar accumulation of up to 100 mM zinc (7 × 10^8^ vacuolar zinc ions per cell) (Simm *et al.* 2007). *ZRC1* and *COT1* are paralogous genes, which likely arose from the whole genome duplication event in the *Saccharomyces* lineage, and many other fungi encode only a single copy of the vacuolar zinc importer. This ‘zinc sink’ can be rapidly mobilized from the vacuole to the cytoplasm via a third ZIP-type transporter, Zrt3 (MacDiarmid, Gaither and Eide 2000), and serve the cell as an effective zinc source when this micronutrient is not present in the extracellular environment. Indeed, Simm *et al.* (2007) demonstrated that a zinc-sated *S. cerevisiae* vacuole could provide a progenitor mother cell with sufficient zinc for eight generations (equivalent to the generation of 200 new cells), in the absence of external zinc. Although vacuolar zinc homeostasis has not yet been investigated in great detail in pathogenic fungi, the generally high levels of zinc present in growth media typically used for virulence assays (e.g. YPD—yeast extract, peptone, glucose) may have significant implications on the ability of pathogenic fungi to grow within mammalian hosts.

The pathogenic mould, *A. fumigatus* encodes orthologues of both ScZrt1 (AfZrfA) and ScZrt2 (AfZrfB), as well as an orthologue of *C. albicans* Zrt1 (AfZrfC), and two as-yet uncharacterized paralogous proteins, ZrfD and Afu8g04010, all five of which are confirmed or predicted cell surface zinc importers (Wilson 2015). As is the case in *S. cerevisiae*, zinc transport in *A. fumigatus* is regulated by ZafA (the *Aspergillus* orthologue of yeast Zap1) in response to environmental zinc availability (Moreno *et al.* 2007). Notably, deletion of *zafA* rendered *A. fumigatus* avirulent in a mouse model of invasive aspergillosis, highlighting the essentiality of coordinated zinc homeostasis during fungal infection.

In contrast, while the regulation of *S. cerevisiae ZRT1* and *ZRT2* has not been reported to be affected by culture pH, or targeted by the Rim101 pathway, *A. fumigatus zrfA*, *zrfB* and *zrfC* expression levels are highly pH regulated, and the three proteins function differentially in acidic and neutral/alkaline environments. As would be expected, this pH-dependent regulation of zinc transport is under control of the PacC transcription factor (PacC is the *Aspergillus* orthologue of yeast Rim101). *zrfA* and *zrfB* are more highly expressed under acidic pH, and deletion of both genes prohibited growth under acidic, zinc-limiting conditions (Vicentefranqueira *et al.* 2005). Notably, a strain lacking both *zrfA* and *zrfB* genes exhibited wild-type levels of virulence in murine infection models (Amich *et al.* 2014). In contrast, deletion of *zrfC* both blocked growth of *A. fumigatus* under zinc depletion at neutral/alkaline pH (Amich *et al.* 2010) and attenuated virulence in mouse models of aspergillosis. These, together with other studies (McDonagh *et al.* 2008) indicate that *A. fumigatus* faces environments of zinc depletion and neutral/alkaline pH during experimental infection.

Zinc transport has recently been investigated in *Cryptococcus gattii*. This Basidiomycete human pathogen encodes two predicted cell surface zinc transporters—Zip1 and Zip2 (Schneider Rde *et al.* 2015). Zip1 shares sequence similarity with *A. fumigatus* ZrfA and ZrfB, whilst *C. gattii* Zip2 is an orthologue of ZrfC and both encoding genes are positively regulated by *C. gattii* Zap1 (Schneider Rde *et al.* 2012). Interestingly, whilst deletion of *C. gattii ZAP1* attenuated virulence in a murine model of cryptococcosis (Schneider Rde *et al.* 2012), single deletions of either *ZIP1* or *ZIP2* had no impact on virulence. However, a double deletion mutant lacking both *ZIP1* and *ZIP2* displayed strongly attenuated virulence (Schneider Rde *et al.* 2015). This study suggests that, in contrast to *A. fumigatus*, the functions of *C. gattii* Zip1 and Zip2 *in vivo* are redundant. Intriguingly, in contrast to *A. fumigatus* and *C. albicans* (see below), *C. gattii* zinc transport does not appear to be pH-dependent, perhaps explaining the observed functional redundancy.

The major human pathogenic yeast, *C. albicans* encodes two predicted cell surface zinc transporters: Zrt1 and Zrt2. Interestingly, despite the fact that *C. albicans* and *S. cerevisiae* are more closely related species (both are members of the Saccharomycotina), regulation of *C. albicans ZRT1* and *ZRT2* appears to be more similar to that of *A. fumigatus*. Whist both genes are targets of Zap1 (also known as Csr1 in *C. albicans*) (Kim *et al.* 2008; Nobile *et al.* 2009), they are also pH regulated. Bensen and co-workers found that *CaZRT1* and *CaZRT2* were differentially upregulated at alkaline and acidic pH, respectively. As *C. albicans* can infect multiple target organs of widely varying pH—from the acidic environment of the vaginal mucosae to neutral/alkaline pH during liver invasion (Thewes *et al.* 2007)—it is possible that Zrt1 and Zrt2 display niche-specific roles during infection (Kumamoto 2008; Wilson *et al.* 2009).

*Candida albicans* Zrt2 likely plays an important role during invasive candidiasis, as overexpression of *ZRT2* in a *sut1*Δ mutant increased the virulence of this strain (Xu *et al.* 2015). Although the role of Zrt1 in virulence has not yet been directly assessed, an interesting function for this transporter has been described. Citiulo *et al.* (2012) found that the secreted protein Pra1 (*p*H-*r*egulated *a*ntigen) was able to bind zinc and sequester this metal from host cells, representing a functional ‘zincophore’ system. Interestingly, *PRA1* was found to share its promoter with *ZRT1* (Nobile *et al.* 2009) and the two genes are coexpressed (Bensen *et al.* 2004). Notably, deletion of *ZRT1* in *C. albicans* prevented the reassociation of soluble Pra1 to the fungal cell, indicating that, in addition to functioning as a zinc transporter (Kim *et al.* 2008; Nobile *et al.* 2009; Wilson 2015), Zrt1 also serves as a receptor for the Pra1 zincophore (Citiulo *et al.* 2012; Wilson, Citiulo and Hube 2012). As the orthologues of *C. albicans* Pra1 in *S. cerevisiae* and *A. fumigatus* (Zps1 and AspF2, respectively) are also pH and zinc regulated, and, in the case of *A. fumigatus*, required for zinc assimilation at alkaline pH, it is tempting to speculate that the zincophore function described for *C. albicans* may be conserved in other fungal species (Wilson 2015).

### Micronutrients take centre stage during host–pathogen interactions

We have discussed the mechanisms of host nutritional immunity and the fungal countermeasures deployed to scavenge micronutrients from host tissues; but why is this interplay so important for the pathogenic outcome of an infection? Micronutrients such as iron and zinc play crucial roles in cellular function. Indeed metalloenzymes are present in all six Enzyme Commission classes, and 47% of enzymes are estimated to require metals for function (Waldron *et al.* 2009). Furthermore, certain non-catalytic proteins (e.g. zinc finger transcription factors) require metals for structure and function. Intriguingly, many fungal proteins which are directly involved in host–pathogen interactions bind, and/or require, metal cofactors. For example, Pra1 has been shown to play multiple immunemodulatory roles, including binding factor H and other components of the complement system (Zipfel *et al.* 2011), and serves as a ligand for neutrophil αMβ2 (Soloviev *et al.* 2007).

Importantly, immune phagocytes including macrophages and neutrophils attempt to kill their prey via the generation of ROS: the ‘oxidative burst’ (Miramon, Kasper and Hube 2013). In turn, fungi express dedicated detoxification mechanisms in order to counteract ROS and survive oxidative stress. Intriguingly, both superoxide dismutases (SODs), which convert superoxide anions to hydrogen peroxide, and catalases, which subsequently detoxify hydrogen peroxide to water and molecular oxygen, require metal cofactors for function. Catalase is an iron-dependent enzyme due to the presence of a haem group, which is essential for the initial reduction of H_2_O_2_ (Hansberg, Salas-Lizana and Dominguez 2012). In contrast, different SOD enzymes utilize different metal cofactors. The model yeast *S. cerevisiae* encodes two SODs: the cytosolic copper/zinc-dependent Sod1 and the mitochondrial, manganese-dependent Sod2 (Leitch, Yick and Culotta 2009). *Candida albicans* also possesses a cytosolic Cu/Zn Sod1 and a pair of mitochondrial manganese-dependent paralogues (Sod2 and Sod3). Intriguingly, this human pathogen encodes a further subfamily of Cu/Zn-like SODs (Sod4,5,6). These lineage-specific SODs are expressed at the fungal cell surface and play a pivotal role in detoxifying ROS generated by immune phagocytes (Dantas Ada *et al.* 2015). Therefore, by combining nutritional immunity with the oxidative killing machinery of the ‘classical’ innate immune system, the host may synergize antifungal activity (Fig. 3). That is, by simultaneously exposing fungal pathogens to oxidative stress, whilst denying them the cofactors required to defend themselves, the immune system may significantly amplify its antimicrobial activity. Indeed, the concept of combinatorial stresses having synergistic effects on fungal killing has recently been demonstrated (Kaloriti *et al.* 2012, 2014).

This central role of metals in resisting immune attack has recently taken an interesting twist. *Candida albicans* Sod5 is a key component of the oxidative stress machinery: *SOD5* expression is induced by oxidative stress *in vitro* and by exposure to human neutrophils (Frohner *et al.* 2009; Miramon *et al.* 2012). Furthermore, deletion of *SOD5* renders *C. albicans* susceptible to killing by human neutrophils and results in virulence attenuation in a murine model of haematogenous disseminated candidiasis (Martchenko *et al.* 2004; Fradin *et al.* 2005; Miramon *et al.* 2012). Therefore, Sod5 plays a pivotal role in *C. albicans* immune evasion and virulence. As zinc is restricted by the action of calprotectin (above), this phenomenon may limit the activity of Sod5. Herein lies the twist: *C. albicans* Sod5 has lost zinc-binding activity and evolved a copper-only cofactor requirement (Gleason *et al.* 2014). In principle, this adaptation would allow pathogenic species to efficiently detoxify superoxide anions, even when zinc is unavailable due to the action of nutritional immunity.

In addition to their essentiality for growth and development, trace metals are also highly toxic when present at elevated concentrations. Interestingly, it has been found that *Cr. neoformans* relies on efficient copper detoxification during pulmonary infections, and it would appear that the host immune system attempts to ‘poison’ the invading fungus with high levels of this metal (Ding *et al.* 2013), a phenomenon which has also been observed for pathogenic bacteria (Neyrolles, Mintz and Catty 2013). *Candida albicans* also requires copper detoxification for virulence (Weissman *et al.* 2000), suggesting that copper toxicity may be a commonly encountered stress during infection. Therefore, our immune systems appear to have harnessed both the essentiality and toxic potential of micronutrients in their battle with pathogenic fungi, and pathogens, in turn have not only had to evolve efficient uptake systems, but also detoxification mechanisms. For a more in-depth review on the phenomenon of metal toxicity in fungal pathogenicity, readers are directed towards the excellent review by García-Santamarina and Thiele (2015).

## OUTLOOK

In summary, the battle for micronutrients represents a fundamental element of the host–pathogen interaction. The action of nutritional immunity serves to limit microbial proliferation by depriving the invading pathogen of essential nutritional resources, and enhancing the activity of other arms of the innate immune system. However, those fungi which have evolved pathogenic potential must also have evolved mechanisms to circumvent the action of nutritional immunity and forage for micronutrients in the restrictive environment of the infected host. This review has focused on the role of iron and zinc during mycoses; however, other metals, such as manganese, likely play crucial roles in the outcome of fungal infections; yet, their role in fungal pathogenesis remains poorly understood. The challenge now will be to understand both host- and pathogen-micronutrient homeostatic mechanisms, how they interact in the context of pathogenesis and how they may be manipulated in order to favour the host.
